# Sex-specific genetic variants associated with adult-onset inguinal hernia in a Taiwanese population

**DOI:** 10.7150/ijms.82331

**Published:** 2023-03-27

**Authors:** Hsin-Chien Yen, I-Chieh Chen, Guan-Cheng Lin, Yu-Yuan Ke, Ming-Chih Lin, Yi-Ming Chen, Chia-Chi Hsu

**Affiliations:** 1 Division of Pediatric Genetics and Metabolism, Children's Medical Center, Taichung Veterans General Hospital, Taichung, Taiwan; 2 Department of Medical Research, Taichung Veterans General Hospital, Taichung, Taiwan; 3 School of Medicine, National Yang Ming Chiao Tung University, Taipei, Taiwan; 4 Department of Post-Baccalaureate Medicine, College of Medicine, National Chung Hsing University, Taichung, Taiwan; 5 Children's Medical Center, Taichung Veterans General Hospital, Taichung, Taiwan; 6 Department of Food and Nutrition, Providence University, Taichung, Taiwan; 7 School of Medicine, Chung Shan Medical University, Taichung, Taiwan; 8 Division of Allergy, Immunology, and Rheumatology, Department of Internal Medicine, Taichung Veterans General Hospital, Taichung, Taiwan; 9 Institute of Biomedical Science and Rong Hsing Research Center for Translational Medicine, National Chung Hsing University, Taiwan

**Keywords:** Inguinal hernia, *WT1 rs3809060*, * EFEMP1* rs2009262, BPH, COPD, BMI

## Abstract

**Introduction:** Inguinal hernia repair is one of the most common surgeries worldwide. However, there is limited information on its underlying genetic mechanism. Studies on the genetic factors related to inguinal hernia in Han Chinese are lacking. Therefore, we aimed to conduct a hospital-based study to assess the genetic factors and comorbidities underlying inguinal hernia in Taiwan.

**Materials and Methods:** This was a retrospective case-control study. Utilizing data from the Taiwan Precision Medicine Initiative, we identified 1000 patients with inguinal hernia and 10,021 matched controls without inguinal hernia between June 2019 and June 2020. Four susceptibility loci (rs2009262, rs13091322, rs6991952, and rs3809060) associated with inguinal hernia were genotyped by the Taiwan Biobank version 2 (TWBv2) array. Inguinal hernia, surgery types, and comorbidities were obtained from the electronic health records of Taichung Veterans General Hospital.

**Results:** Adult-onset inguinal hernia was associated with *WT1* rs3809060 GT/TT genotype in males and *EFEMP1* rs2009262 TC/CC genotype in females. In addition, we identified sex-specific risk factors associated with inguinal hernia; benign prostatic hyperplasia in males (OR: 3.19, 95% CI: 2.73 - 3.73, *p*< 0.001), chronic obstructive pulmonary disease in females (OR: 2.34, 95% CI: 1.33 - 4.11, p = 0.003) and overweight, defined by body mass index ≧24 kg/m^2^ (OR: 0.75, 95% CI: 0.65 - 0.86, *p*<0.001 in males, and OR: 0.60, 95% CI:0.37 - 0.98, *p* = 0.042 in females), were inversely associated with inguinal hernia.

After stratifying BMI, overweight males with *EFEMP1* rs2009262 TC/CC genotype exhibited a higher risk of inguinal hernia (OR: 1.31, 95% CI: 1.07 - 1.61, p = 0.01). Additionally, rs3809060 was specifically associated with male patients with direct-type inguinal hernia (OR: 1.62, 95% CI: 1.19 - 2.22, *p* = 0.002).

**Conclusion:** Genetic susceptibility appears to participate in the pathogenesis of inguinal hernia in the Taiwanese population in a sex-specific manner. Future studies are needed to illuminate the complex interplay between heredity and comorbidities.

## Introduction

Inguinal hernia is the consequence of protrusion of abdominal viscus out of the lining of the abdominal cavity through a weak spot in the transversalis and oblique tissues of the low abdominal wall.^1^ An epidemiological investigation revealed an estimated prevalence of 4.36% in the Americas, 4.06% in Europe, and 4.88% in Southeast Asia.^2^ Men are reported to have a much higher lifetime prevalence of inguinal hernias (20-27%) than women (3-6%).^3^ In terms of anatomical location, inguinal hernias are divided into either direct or indirect types. Direct inguinal hernias bulge medially toward the inferior epigastric vessels, due to an acquired defect in the fibromuscular tissues of the inguinal canal, and indirect inguinal hernia sacs protrude laterally toward the inferior epigastric artery, due to late closure of the processus vaginalis.

Inguinal hernias have a variety of symptoms ranging from a painless lump in the groin area to serious complications such as strangulation and bowel incarceration, and surgical repair is currently the only definitive treatment. The rate of recurrent inguinal hernia was reported to range from 0.5 to 15 percent, depending upon the type of repair, hernia site, and the clinical circumstances.^4^ Multiple risk factors for the formation of inguinal hernia have been recognized, including older age, male sex,^5^ lower body mass index,^6^ patent processus vaginalis,^7^ matrix metalloproteinase (MMP) abnormalities,^1^ chronic obstructive pulmonary disease,^8^ and history of prostatectomy.^9^ Greater body height and rural residence were also reported to be associated with direct inguinal hernia in females.^3^ Although male sex is a well-known predictor for inguinal hernia, it remains unclear whether a sex-specific risk factor exists, based on prior studies.

Family history is a strong risk factor for inguinal hernia.^10^, ^11^ Susceptible genes associated with inguinal hernia have been reported,^12^-^15^ suggesting heredity may be conducive to the pathogenesis of inguinal hernias. Jorgenson et al. conducted the first genome-wide association study and recognized four significant inguinal hernia risk loci (EBF2, ADAMTS6, EFEMP1 and WT1).^16^ It has been demonstrated that these four genes contribute to elastin maintenance and collagen equilibrium and are involved in the development of inguinal hernia. Genetic heterogeneity may exist among different ethnicities, but the majority of previous genetic studies were conducted in Caucasian populations. The risk loci associated with inguinal hernia in Asians remain unclear.

The primary aim of this study was to verify whether susceptibility loci (rs2009262, rs13091322, rs6991952, and rs3809060) previously reported in Europeans^16^, ^17^ contribute to inguinal hernia and comorbidities in the Taiwanese population. The secondary aim was to distinguish sex-specific risk factors for the development of inguinal hernia in a hospital-based cohort.

## Material and Methods

### Study Design and Data Source

This was a retrospective case-control study. The data source was the Taiwan Precision Medicine Initiative (TPMI), a countrywide genetic scheme overseen by Academia Sinica and partner hospitals. The TPMI cohort consisted of Taiwanese participants in 16 hospitals across the country, from whom specimens and information were collected, as well as patients at Taichung Veterans General Hospital (TCVGH), a tertiary medical center, who comprised the greater part of the cohort. A total of 58,091 patients aged >18 years who visited 28 surgical and medical outpatient clinics in TCVGH were selected for enrollment in the TPMI study between June 2019 and June 2020. Our study cohort comprised 11021 patients, whose genetic profiles were connected to medical claims data in TCVGH, including demographic characteristics, procedures, examinations, diagnoses, medication prescriptions, surgeries, outpatient services, and inpatient services. Diagnoses are coded employing the International Classification of Diseases, Ninth Revision, Clinical Modification (ICD-9-CM) format. This study protocol was approved by the institutional review board of Taichung Veterans General Hospital (IRB no. SF19153A). All of the participants provided informed consent.

### Case identification

**Figure 1:**
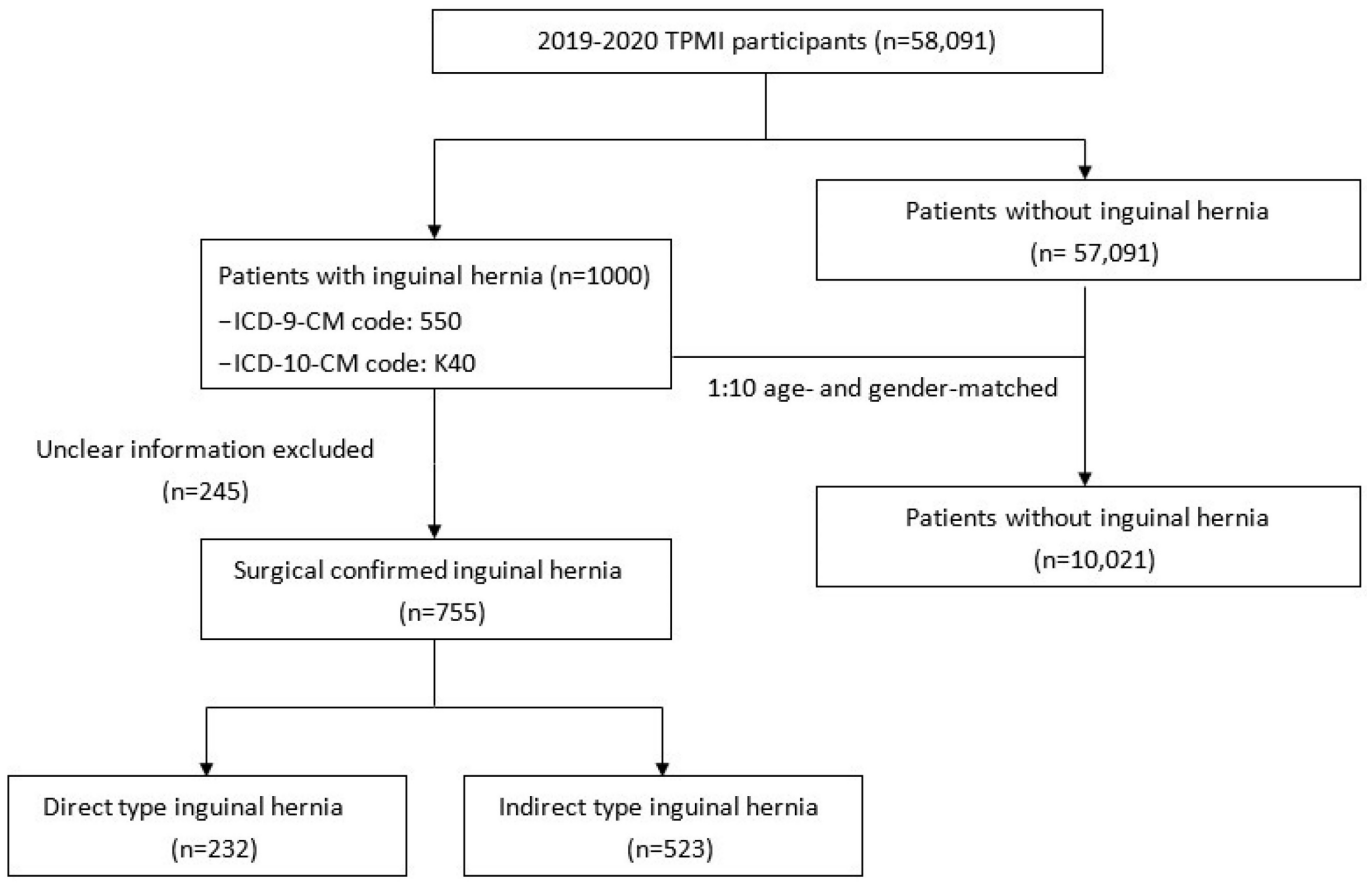
Flow chart of study design.

A total of 1,000 patients who had a clinical diagnosis of inguinal hernia (ICD-9-CM code 550, ICD-10-CM code K40) during hospitalization or in an outpatient clinic were selected as the case group (Figure 1). Among them, 755 had available surgery records in TCVGH with postoperative diagnosis of direct or indirect inguinal hernia. A total of 10,021 patients without a medical history of inguinal hernia were matched to the case group by gender and age at a ratio of 1:10 to serve as the control group. We obtained comorbidity information from the electronic health records of TCVGH based on ICD-9 diagnostic codes. Comorbidities of hyperlipidemia (ICD-9-CM code 272, ICD-10-CM code E78.1-E78.5), hypertension (ICD-9-CM code 401-405, ICD-10-CM code I10-I15), diabetes mellitus (ICD-9-CM code 250, ICD-10-CM code E10.9 and E11.9), benign prostatic hyperplasia (BPH) (ICD-9-CM code 600.2, ICD-10-CM code N40.0), chronic kidney disease (CKD) (ICD-9-CM code 585 and ICD-10 code N18.1-N18.9), and chronic obstructive pulmonary disease (COPD) (ICD-9 code 491, ICD-10-CM code J44) were identified if the diagnostic code was used once during admission or at least twice in the outpatient service.

### Genotyping

In our study, genomic DNA was extracted from 58,091 TPMI participants using DNA isolation kits (TIANGEN Biotech, Beijing, China) and quantified using a NanoDrop 2000 Spectrophotometer (NanoDrop Technologies, Wilmington, DE, USA). Then, the Taiwan Biobank version 2 (TWBv2) array was used to perform next generation sequencing (NGS) for the participants, including 114,000 risk variants in 2,831 unusual disease genes selected from ClinVar, ACMG, GWAS Catalog, HGMD, locus-specific databases, and the published literature.^18^ After literature review, we chose four available single nucleotide polymorphisms (SNP), namely, rs2009262, rs13091322, rs6991952, and rs3809060, for further analyses, which were previously reported by Jorgenson et al.^16^ and Hikino et al.^17^ Quality control of genotyping included an exclusion of SNPs if only one allele appeared in the study cohort; the total call rate < 95% or the total MAF < 0.01. In addition, SNPs significantly departing from the Hardy-Weinberg equilibrium (P < 1 × 10-4) were also excluded. PLINK 1.9 was utilized for statistical genetic analyses and quality control of genetic data.

### Statistics

We analyzed all data using the Statistical Package for the Social Sciences (SPSS, IBM Corp., Armonk, NY, USA) version 22.0. The baseline demographics are expressed as number (percent) for categorical variables and the mean ± standard deviation (SD) for continuous variables. Chi-square test for categorical variables and Student's t-test for continuous variables were utilized to compare variables between inguinal hernia cases and controls. Univariate and multivariate logistic regression analyses were applied to estimate the associations of genotypes with the risks of hernia. Odds ratios (ORs) and 95% confidence intervals (95% CIs) were calculated. Significant covariates were contained in the final model and a 2-sided *p* value of < 0.05 was interpreted as statistically significant in this study.

## Results

### Baseline characteristics and genetic variations associated with inguinal hernia

The baseline demographics are illustrated in Table 1. Inguinal hernia developed mostly at the age of > 65 years (67.9%), with significant male dominance (90.5%). Participants with inguinal hernia exhibited higher percentages of BPH (60% vs. 36.8%, *p* < 0.001), and COPD (23.8% vs. 19.4%, *p* = 0.001). Overweight (BMI ≧ 24 kg/m^2^) (45.5% vs. 54.1%, *p* < 0.001), hyperlipidemia (40.2% vs. 45.5%, *p* = 0.001), diabetes mellitus (30.9% vs. 43.9%, *p* < 0.001), and CKD (31.0% vs. 34.4%, *p* = 0.04) were inversely associated with inguinal hernia.

The genotype frequencies of the four susceptibility loci (rs2009262, rs13091322, rs6991952, and rs3809060) are shown in Table 2. A greater prevalence of the *EFEMP1* rs2009262 TC/CC genotype (36.6% vs. 32.6%, *p* = 0.01) and the *WT1* rs3809060 GT/TT genotype (48.1% vs. 44.1%, *p* = 0.02) was found in the inguinal hernia group than in the control group.

### Gender differences in the onset age of inguinal hernia

**Figure 2:**
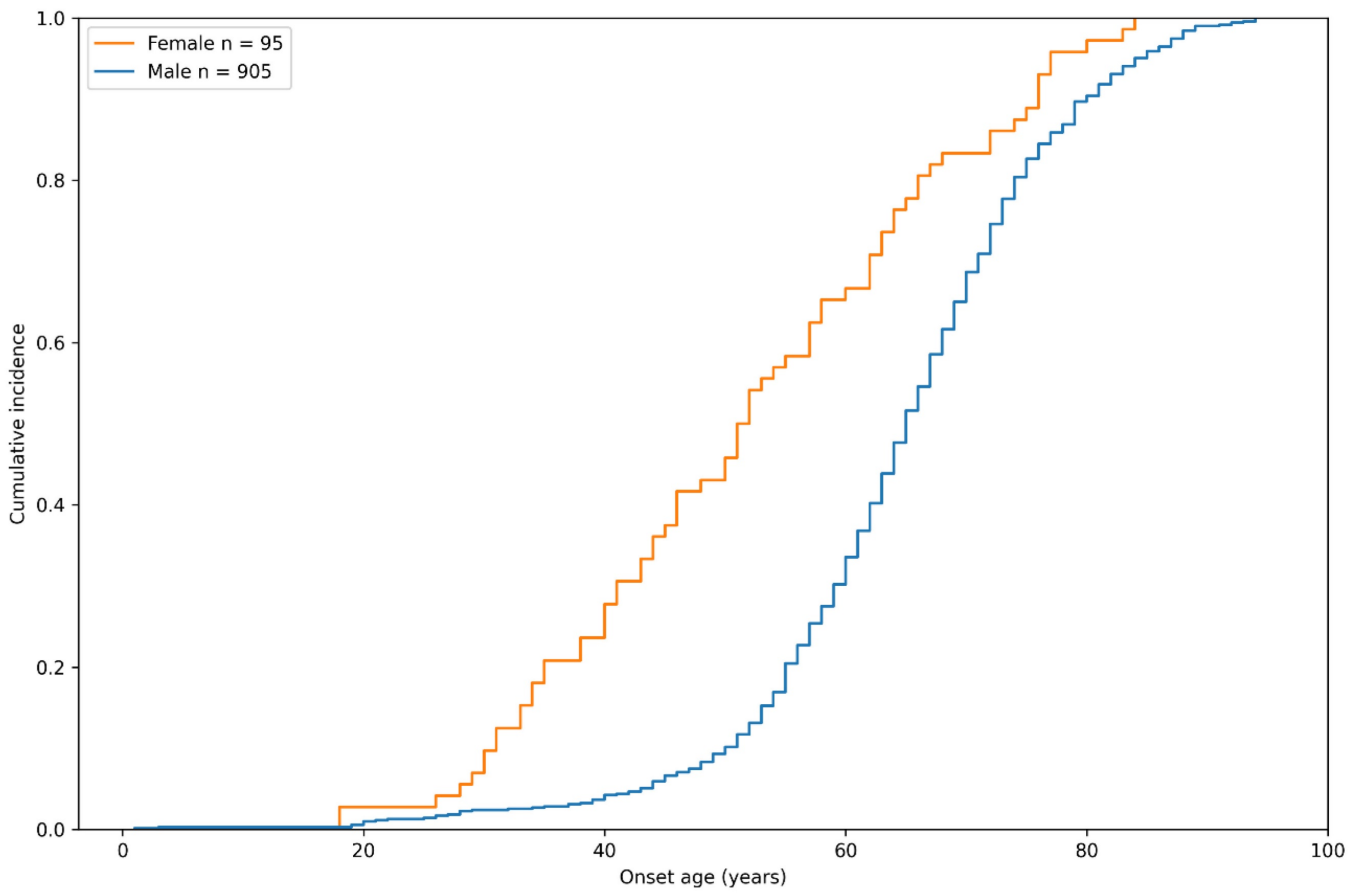
Sex-specific cumulative incidence curve of inguinal hernia in the study group by onset age.

The sex-specific cumulative incidence of inguinal hernia in the case group of the study population is depicted in Figure 2. Interestingly, we found that although there was a higher prevalence of male patients with inguinal hernia, female participants seemed to develop this disease at an earlier age. By the age of 60 years, 66.7% of female participants developed inguinal hernia, compared to only 33.6% of males, indicating that the pathogenesis of hernia might be different between genders.

### Sex-specific factors associated with inguinal hernia

In order to investigate sex-specific risk factors for inguinal hernia, univariate analysis was performed (Table 3). In the female group, the *EFEMP1* rs2009262 TC/CC genotype was significantly associated with inguinal hernia (OR: 1.71, 95% CI: 1.12-2.62, *p* = 0.01). Comparatively, in males with inguinal hernia, *WT1* rs3809060 GT/TT was found to be a genetic variation significantly correlated with inguinal hernia (OR: 1.17, 95% CI: 1.02-1.35, *p* = 0.02). Both males and females in the inguinal hernia group had a lower BMI as compared to their counterparts.

In the multivariate analysis of males (Table 4), genotype *WT1* rs3809060 GT/TT and BPH (OR: 3.19, 95% CI: 2.73 - 3.73, *p* < 0.001) were independently associated with a higher risk of inguinal hernia, while diabetes mellitus (OR: 0.61, 95% CI: 0.52 - 0.72, *p* < 0.001) was negatively associated with development of inguinal hernia. In the female group, genotype* EFEMP1* rs2009262 TC/CC and COPD (OR: 2.34, 95% CI: 1.33 - 4.11, *p* = 0.003) were associated with greater incidence of inguinal hernia. The risk of inguinal hernia was significantly inversely associated with BMI **≧** 24 kg/m^2^ in men (OR: 0.75, 95% CI: 0.65 - 0.86, *p*<0.001) and women (OR: 0.60, 95% CI:0.37 - 0.98, *p*=0.042), compared with their counterparts.

### BMI and genetic variants in different genders

We further stratified participants based on sex and BMI levels to explored the association of the genetic variants and overweight (BMI ≧ 24 kg/m^2^) with inguinal hernia. We conducted a multivariate logistic regression analysis as shown in Table 5, overweight males with* EFEMP1* rs2009262 variant had a higher risk of inguinal hernia development after adjusted for potential confounders (OR: 1.31, 95% CI: 1.07-1.61, p = 0.010). Conversely, among females with BMI < 24 kg/m^2^, carriers of *EFEMP1* rs2009262 had a 1.83-fold increased risk of inguinal hernia (OR: 1.83, 95% CI: 1.10-3.05, p = 0.02).

### Genotypes and inguinal hernia types

To further delineate genetic variation associated with hernia types, a multivariate regression analysis was conducted (Table 6). We identified that rs3809060 was significantly correlated with direct type inguinal hernia in males (OR: 1.62, 95% CI: 1.19 - 2.22, *p* = 0.002).

## Discussion

This hospital-based case-control study identified preferential SNPs with increased susceptibility to adult-onset inguinal hernia; *EFEMP1* rs2009262 in females and *WT1* rs3809060 in males, specifically in association with direct-type inguinal hernia. We also demonstrated sex-specific risk factors associated with inguinal hernia, with COPD in women, BPH in men, and BMI in both genders. The *WT1* rs3809060 locus exhibited an association with hernia risk among male participants, while overweight (BMI ≧ 24 kg/m^2^) males carrying the TC/CC genotype of the *EFEMP1* rs2009262 variant were found to be at an increased risk of developing inguinal hernia. This study is the first to demonstrate genetic susceptibility loci of inguinal hernia in a Han Chinese population. Our result indicates that heredity plays a compelling role in the pathogenesis of inguinal hernia in a sex-specific manner.

Genetic defects in any components of the inguinal canal, including collagen, microfibrils, elastin, abdominal wall muscles, and glycosaminoglycan elements of the extracellular matrix, could result in inguinal hernia formation.^19^ A number of genes or risk loci associated with inguinal hernia were identified by candidate gene approach^12^ and genome-wide association studies;^16^, ^17^, ^20^ most of them were correlated with connective tissue homoeostasis, such as elastin (ELN).^17^ Decreased tissue concentration of ELN was observed in inguinal hernia patients,^21^ and it has been reported that variants in *ELN* gene are associated with inguinal hernia development.^20^ Epidermal growth factor-containing fibulin-like extracellular matrix protein 1 (*EFEMP1*) belongs to the fibulin gene family, and it is crucial for ELN production and elastic fiber formation by regulating incorporation of elastin to the microfibril architecture.^22^ *EFEMP1* knockout mice present diminished elastic fibers in fascia and appear to have inguinal hernias or other large hernias.^23^ *EFEMP1* variants are also associated with various functional alterations, including body height,^24^ waist circumference,^25^ and forced vital capacity.^26^ These collective correlations indicate that abnormal elastin tissue may result in the development of both inguinal hernia and COPD, which is compatible with our finding that COPD was associated with greater incidence of inguinal hernia in the female group.

Additionally, the altered activity of tissue inhibitor of metalloproteinases (TIMPs) and collagen degrading matrix metalloproteinases (MMPs) led to distorted collagen metabolism and diminished ratio of type I to type III collagen, which could affect the development of inguinal hernia.^27^, ^28^ Previous studies have reported increased tissue expression of MMP2 and MMP9, while TIMP1 and TIMP3 were decreased in inguinal hernia patients.^28^-^31^ WT1 has been revealed to activate TIMP3 and downregulate MMP-2 and MMP-9,^32^ which could further correlate with dysregulation of the proportion of type I/III collagen and inguinal hernia formation. In men, there are several reasons that could explain the association of BPH and higher risk of inguinal hernia. Significant relations were reported among BPH and prostate cancer as BPH could augment risk of prostate cancer.^33^ WT1 also regulates the levels of E-cadherin, contributing to the migratory potential of prostate cancer cells.^34^ In addition, MMP-9, which is downregulated by WT1,^32^ has been identified as a potential prostate tumor marker, while overexpression of MMP-9 was observed in prostate cancers.^35^ Our result resembles the finding of a previous epidemiological study that showed BPH was associated with later inguinal hernia formation.^36^

In our study, indirect inguinal hernia accounted for 65 percent of repairs in men, which was similar to a previous study in Taiwan.^37^ The OR exhibited for direct-type inguinal hernia was stronger for males with the rs3809060 GT/TT genotype than for the indirect-type inguinal hernia, indicating different underlying mechanisms and genetic susceptibility among the subtypes of inguinal hernia.^38^ In females, as there were few cases with surgically identified inguinal hernia (*N* = 52), no genotype association was nominally observed. We also demonstrated that normal body mass index (BMI<24 kg/m^2^) was inversely related to the formation of inguinal hernia, which has been reported in many previous studies.^39^-^41^ While *EFEMP1* locus has been associated with anthropometric measures of body height and abdominal circumference, shared genetic effects may explain the plausible causality between BMI and inguinal hernia, as previously noted by Hélène Choquet et al.^20^ However, further investigation of different pathophysiological associations of *EFEMP1* in sex-specific manner may be considered as opposite correlations between *EFEMP1* rs2009262 TC/CC and inguinal hernia were observed in different genders according to BMI levels.

There were some limitations in this study. First, we utilized the SNP array for genotyping. Not every variant associated with inguinal hernia was included in the probe design^18^. Hence, it is highly likely that coexistent genetic variants in the study group were not identified. Further study with next-generation sequencing is required to decode genetic contributions to inguinal hernia. Second, our study enrolled only cases with adult-onset inguinal hernia. As the age of inguinal hernia occurrence also peaks in the newborn period due to late closure of the processus vaginalis, it remains unknown whether rs2009262 and rs3809060 could also contribute to the development of pediatric inguinal hernia. Third, our participants were all Taiwanese, and therefore it might not be possible to extrapolate these results to other ethnic groups. Lastly, as our study is hospital-based, only patients who visited outpatient clinics with blood tests participated. The selection bias is likely. Therefore, we may underestimate the effect of these sex-specific risk factors associated with inguinal hernia.

## Conclusion

In summary, this hospital-based study demonstrated a gender difference in the genetic susceptibility of adult-onset inguinal hernia. Further studies are needed to decipher the mechanisms underlying the complex interaction among sex, genetic background, and comorbidities.

## Figures and Tables

**Table 1:** Baseline characteristics of the participants.

Characteristics	Non-inguinal hernia group (n=10,021)	Inguinal hernia group (n=1,000)	*p* value
	n (%)	n (%)	
Age (years)			0.93
< 45	552 (5.5)	57 (5.7)	
45-65	2694 (26.9)	264 (26.4)	
> 65	6775 (67.7)	679 (67.9)	
Gender			1.00
Female	957 (9.5)	95 (9.5)	
Male	9064 (90.5)	905 (90.5)	
Overweight (BMI≧24 kg/m^2^)			< 0.001
No	4603 (45.9)	545 (54.5)	
Yes	5418 (54.1)	455 (45.5)	
Hyperlipidemia			0.001
No	5460 (54.5)	598 (59.8)	
Yes	4561 (45.5)	402 (40.2)	
Hypertension			0.295
No	4801 (47.9)	496 (49.6)	
Yes	5220 (52.1)	504 (50.4)	
Diabetes mellitus			< 0.001
No	5623 (56.1)	691 (69.1)	
Yes	4398 (43.9)	309 (30.9)	
Chronic kidney disease			0.04
No	6577 (65.6)	690 (69.0)	
Yes	3444 (34.4)	310 (31.0)	
BPH			< 0.001
No	6336 (63.2)	400 (40.0)	
Yes	3685 (36.8)	600 (60.0)	
COPD			0.001
No	8072 (80.6)	762 (76.2)	
Yes	1949 (19.4)	238 (23.8)	

Abbreviations: BMI: Body mass index; BPH: benign prostatic hyperplasia; COPD: chronic obstructive pulmonary disease

**Table 2:** Allele frequencies and genetic association with inguinal hernia

Gene / SNP	Non- inguinal hernia group (n=10,021)	Inguinal hernia group (n=1,000)	*p* value
	n (%)	n (%)	
*EFEMP1* rs2009262			0.01
TT	6756 (67.4)	634 (63.4)	
TC/CC	3265 (32.6)	366 (36.6)	
*WT1* rs3809060			0.02
GG	5598 (55.9)	519 (51.9)	
GT/TT	4423 (44.1)	481 (48.1)	
*ERC2* rs13091322			0.72
AA	6416 (64.0)	634 (63.4)	
AG/GG	3605 (36.0)	366 (36.6)	
*EBF2* rs6991952			0.38
AA	6101 (60.9)	594 (59.4)	
AG/GG	3920 (39.1)	406 (40.6)	

Abbreviations: SNP: Single Nucleotide Polymorphism

**Table 3:** Sex-stratified risk of inguinal hernia with *EFEMP1* rs2009262 and *WT1* rs3809060

	Male				
Variables	Non- inguinal hernia group (n=9,064)	Inguinal hernia group (n=905)	*P value*	Risk of Hernia	
	n (%)	n (%)		OR (95%CI)	*p* value
Age, years (%)					
<45	303 (3.3)	32 (3.5)		1	
45-65	2339 (25.8)	230 (25.4)		0.93 (0.63-1.37)	0.72
>65	6422 (70.9)	643 (71.0)	0.93	0.95 (0.65-1.38)	0.78
Overweight (BMI ≧ 24 kg/m^2^)					
No Yes	4035 (44.5) 5029 (55.5)	476 (52.6) 429 (47.4)	<0.001	1 0.72 (0.63-0.83)	< 0.001
*EFEMP1* rs2009262					
TT	6111 (67.4)	582 (64.3)		1	
TC/CC	2953 (32.6)	323 (35.7)	0.06	1.15 (0.99-1.32)	0.06
*WT1* rs3809060					
GG	5068 (55.9)	470 (51.9)		1	
GT/TT	3996 (44.1)	435 (48.1)	0.02	1.17 (1.02-1.35)	0.02
	Female				
Age, years (%)					
<45	249 (26.0)	25 (26.3)		1	
45-65	355 (37.1)	34 (35.8)		0.95 (0.55-1.64)	0.86
>65	353 (36.9)	36 (37.9)	0.97	1.01 (0.59-1.73)	0.95
Overweight (BMI ≧ 24 kg/m^2^)					
No Yes	568 (59.4) 389 (40.6)	69 (72.6) 26 (27.4)	0.016	1 0.55 (0.34-0.88)	0.01
*EFEMP1* rs2009262					
TT	645 (67.4)	52 (54.7)		1	
TC/CC	312 (32.6)	43 (45.3)	0.02	1.71 (1.12-2.62)	0.01
*WT1* rs3809060					
GG	530 (55.4)	49(51.6)		1	
GT/TT	427 (44.6)	46 (48.4)	0.55	1.17 (0.76-1.78)	0.48

**Table 4:** Multivariate logistic regression analysis of risks associated with inguinal hernia by sex^a^

Variables	Male			Female		
	OR	95% CI	*p* value	OR	95% CI	*p* value
*EFEMP1* rs2009262 TC/CC	1.14	0.99-1.32	0.072	1.74	1.13-2.68	0.013
*WT1* rs3809060 GT/TT	1.15	1.00-1.33	0.044	1.23	0.79-1.89	0.347
Overweight (BMI≧24 kg/m^2^)	0.75	0.65-0.86	< 0.001	0.60	0.37-0.98	0.042
Hyperlipidemia	0.94	0.80-1.10	0.409	0.63	0.36-1.10	0.107
Hypertension	1.12	0.96-1.31	0.165	0.81	0.45-1.46	0.479
Diabetes mellitus	0.61	0.52-0.72	< 0.001	0.73	0.40-1.31	0.287
Chronic kidney disease	0.87	0.74-1.02	0.086	0.75	0.42-1.35	0.340
COPD	1.13	0.96-1.34	0.147	2.34	1.33-4.11	0.003
BPH	3.19	2.73-3.73	< 0.001	-	-	-

^a^Model adjusted for genotypes, age, Body mass index, hyperlipidemia, hypertension, diabetes mellitus, chronic kidney disease, chronic obstructive pulmonary disease, and benign prostate hyperplasia. Abbreviations: CI: confidence interval; OR: odds ratio; BMI: Body mass index; BPH: benign prostatic hyperplasia; COPD: chronic obstructive pulmonary disease.

**Table 5:** Sex-specific associations of genetic variants and BMI levels with inguinal hernia^b^

Variables	Male					
	BMI < 24 kg/m^2^			BMI ≧ 24 kg/m^2^		
	OR	95% CI	*p* value	OR	95% CI	*p* value
Age, years	0.98	0.98-0.99	< 0.001	0.99	0.98-0.10	0.002
*EFEMP1* rs2009262 TC/CC	1.00	0.81-1.23	0.997	1.31	1.07-1.61	0.010
*WT1* rs3809060 GT/TT	1.21	0.99-1.46	0.056	1.11	0.91-1.36	0.309
	Female					
Age, years	1.01	0.99-1.02	0.430	1.03	1.00-1.06	0.097
*EFEMP1* rs2009262 TC/CC	1.83	1.10-3.05	0.020	1.44	0.62-3.36	0.395
*WT1* rs3809060 GT/TT	1.32	0.80-2.20	0.281	0.99	0.44-2.27	0.989

^b^Model adjusted for age, genotypes, hyperlipidemia, hypertension, diabetes mellitus, chronic kidney disease, chronic obstructive pulmonary disease, and benign prostate hyperplasia. Abbreviations: CI: confidence interval; OR: odds ratio; BMI: Body mass index; BPH: benign prostatic hyperplasia; COPD: chronic obstructive pulmonary disease.

**Table 6:** Sex-specific genetic variations associated with different inguinal hernia subtypes^c^

SNP	Type	Male		Female	
		OR (95%CI)	*p* value	OR (95%CI)	*p* value
rs2009262					
	Direct	1.13 (0.82-1.55)	0.46	0.68 (0.17-2.77)	0.59
	Indirect	0.89 (0.68-1.17)	0.41	0.75 (0.30-1.92)	0.55
rs3809060					
	Direct	1.62 (1.19-2.22)	0.002	3.35 (0.71-15.69)	0.13
	Indirect	0.78 (0.60-1.02)	0.07	0.55 (0.21-1.43)	0.22

^c^Model adjusted for genotypes, hyperlipidemia, hypertension, chronic kidney disease, diabetes mellitus, chronic obstructive pulmonary disease, benign prostate hyperplasia. Abbreviations: CI: confidence interval; OR: odds ratio.
